# Mindfulness Meditation for Chronic Pain: Systematic Review and Meta-analysis

**DOI:** 10.1007/s12160-016-9844-2

**Published:** 2016-09-22

**Authors:** Lara Hilton, Susanne Hempel, Brett A. Ewing, Eric Apaydin, Lea Xenakis, Sydne Newberry, Ben Colaiaco, Alicia Ruelaz Maher, Roberta M. Shanman, Melony E. Sorbero, Margaret A. Maglione

**Affiliations:** RAND Corporation, 1776 Main Street, PO Box 2138, Santa Monica, CA 90407-2138 USA

**Keywords:** Chronic pain, Mindfulness, Meditation, Systematic review

## Abstract

**Background:**

Chronic pain patients increasingly seek treatment through mindfulness meditation.

**Purpose:**

This study aims to synthesize evidence on efficacy and safety of mindfulness meditation interventions for the treatment of chronic pain in adults.

**Method:**

We conducted a systematic review on randomized controlled trials (RCTs) with meta-analyses using the Hartung-Knapp-Sidik-Jonkman method for random-effects models. Quality of evidence was assessed using the GRADE approach. Outcomes included pain, depression, quality of life, and analgesic use.

**Results:**

Thirty-eight RCTs met inclusion criteria; seven reported on safety. We found low-quality evidence that mindfulness meditation is associated with a small decrease in pain compared with all types of controls in 30 RCTs. Statistically significant effects were also found for depression symptoms and quality of life.

**Conclusions:**

While mindfulness meditation improves pain and depression symptoms and quality of life, additional well-designed, rigorous, and large-scale RCTs are needed to decisively provide estimates of the efficacy of mindfulness meditation for chronic pain.

**Electronic supplementary material:**

The online version of this article (doi:10.1007/s12160-016-9844-2) contains supplementary material, which is available to authorized users.

## Introduction

Chronic pain, often defined as pain lasting longer than 3 months or past the normal time for tissue healing [1], can lead to significant medical, social, and economic consequences, relationship issues, lost productivity, and larger health care costs. The Institute of Medicine recognizes pain as a significant public health problem that costs our nation at least $560–635 billion annually, including costs of health care and lost productivity [2]. Further, chronic pain is frequently accompanied by psychiatric disorders such as pain medication addiction and depression that make treatment complicated [3]. The high prevalence and refractory nature of chronic pain, in conjunction with the negative consequences of pain medication dependence, has led to increased interest in treatment plans that include adjunctive therapy or alternatives to medication [4]. One such modality that pain patients are using is mindfulness meditation. Based on ancient Eastern meditation practices, mindfulness facilitates an attentional stance of detached observation. It is characterized by paying attention to the present moment with openness, curiosity, and acceptance [5, 6]. Mindfulness meditation is thought to work by refocusing the mind on the present and increasing awareness of one’s external surroundings and inner sensations, allowing the individual to step back and reframe experiences. Current research using neuroimaging to elucidate neurological mechanisms underlying effects of mindfulness has focused on brain structures such as the posterior cingulate cortex, which appear to be involved in self-referential processing [7, 8]. Clinical uses of mindfulness include applications in substance abuse [9], tobacco cessation [10], stress reduction [11], and treatment of chronic pain [12–14].

Early mindfulness studies in pain patients showed promising outcomes on pain symptoms, mood disturbance, anxiety, and depression, as well as pain-related drug utilization [5]. Numerous systematic reviews on the effects of mindfulness meditation have been published in recent years. Of those that report pain outcomes, several have focused on specific types of pain such as low back pain [13], fibromyalgia [15], or somatization disorder [16]. Others were not limited to RCTs [14, 17]. There have been several comprehensive reviews focused on controlled trials of mindfulness interventions for chronic pain including a review [4] that showed improvements in depressive symptoms and coping, another review [18] on mindfulness for chronic back pain, fibromyalgia, and musculoskeletal pain that showed small positive effects for pain, and the most recent review [19] on various pain conditions which found improvements in pain, pain acceptance, quality of life, and functional status. Authors of these reviews echoed concerns that there is limited evidence for efficacy of mindfulness-based interventions for patients with chronic pain because of methodological issues. They have concluded that additional high-quality research was needed before a recommendation for the use of mindfulness meditation for chronic pain symptoms could be made.

The purpose of this study was to conduct a systematic review and meta-analysis of the effects and safety of mindfulness meditation, as an adjunctive or monotherapy to treat individuals with chronic pain due to migraine, headache, back pain, osteoarthritis, or neuralgic pain compared with treatment as usual, waitlists, no treatment, or other active treatments. Pain was the primary outcome, and secondary outcomes included depression, quality of life, and analgesic use. The systematic review protocol is registered in an international registry for systematic reviews (PROSPERO 2015:CRD42015025052).

## Methods

### Search Strategy

We searched the electronic databases PubMed, Cumulative Index to Nursing and Allied Health Literature (CINAHL), PsycINFO, and Cochrane Central Register of Controlled Trials (CENTRAL) for English-language-randomized controlled trials from inception through June 2016. We combined pain conditions and design terms with the following mindfulness search terms: “Mindfulness” [Mesh]) or “Meditation” [Mesh] or mindfulness* or mindfulness-based or MBSR or MBCT or M-BCT or meditation or meditat* or Vipassana or satipaṭṭhāna or anapanasati or Zen or Pranayama or Sudarshan or Kriya or zazen or shambhala or buddhis*.” In addition to this search and the reference mining of all included studies identified through it, we reference mined prior systematic reviews and retrieved all studies included therein.

### Eligibility Criteria

Parallel group, individual or cluster RCTs of adults who report chronic pain were included. Studies where the author defined chronic pain and studies in patients reporting pain for a minimum of 3 months were included. Studies were required to involve mindfulness meditation, either as an adjunctive or monotherapy; studies testing other meditation interventions such as yoga, tai chi, qigong, and transcendental meditation techniques without reference to mindfulness were excluded. Mindfulness interventions that did not require formal meditation, such as acceptance and commitment therapy (ACT) were also excluded. Only studies that reported pain measures or change in analgesic use were included. Dissertations and conference abstracts were excluded.

### Procedures

Two independent reviewers screened titles and abstracts of retrieved citations—following a pilot session to ensure similar interpretation of the inclusion and exclusion criteria. Citations judged as potentially eligible by one or both reviewers were obtained as full text. The full text publications were then dually screened against the specified inclusion criteria. The flow of citations throughout this process was documented in an electronic database, and reasons for exclusion of full-text publications were recorded. Data abstraction was also conducted in dual. Risk of bias was assessed using the Cochrane Risk of Bias tool [20]. Other biases related to the US Preventive Services Task Force’s (USPSTF) criteria for internal validity of included studies were assessed [21, 22]. These criteria were used to rate the quality of evidence as good, fair, or poor for each included study.

### Meta-analytic Techniques

When sufficient data were available and statistical heterogeneity was below agreed thresholds [20], we performed meta-analysis to pool efficacy results across included studies for the outcomes of interest and present a forest plot for the main meta-analysis. We used the Hartung-Knapp-Sidik-Jonkman method for random effects meta-analysis using unadjusted means and measures of dispersion [23–25]. For studies reporting multiple pain outcomes, we used specific pain measures, such as the McGill Pain Questionnaire (MPQ) for the main meta-analysis rather than the pain subscale of the SF-36, and average or general pain measures rather than situational measures such as pain at the time of assessment. Due to the small number of adverse events reported, quantitative analysis was not conducted. We conducted subgroup analyses and meta-regressions to address whether there were differences in effect sizes between different interventions types, populations, or when used as monotherapy versus an adjunctive therapy. The quality of the body of evidence was assessed using the GRADE approach [22, 26] by which a determination of high, moderate, low, or very low was made for each major outcome [27].

## Results

### Description of Included Studies

**Fig. 1:**
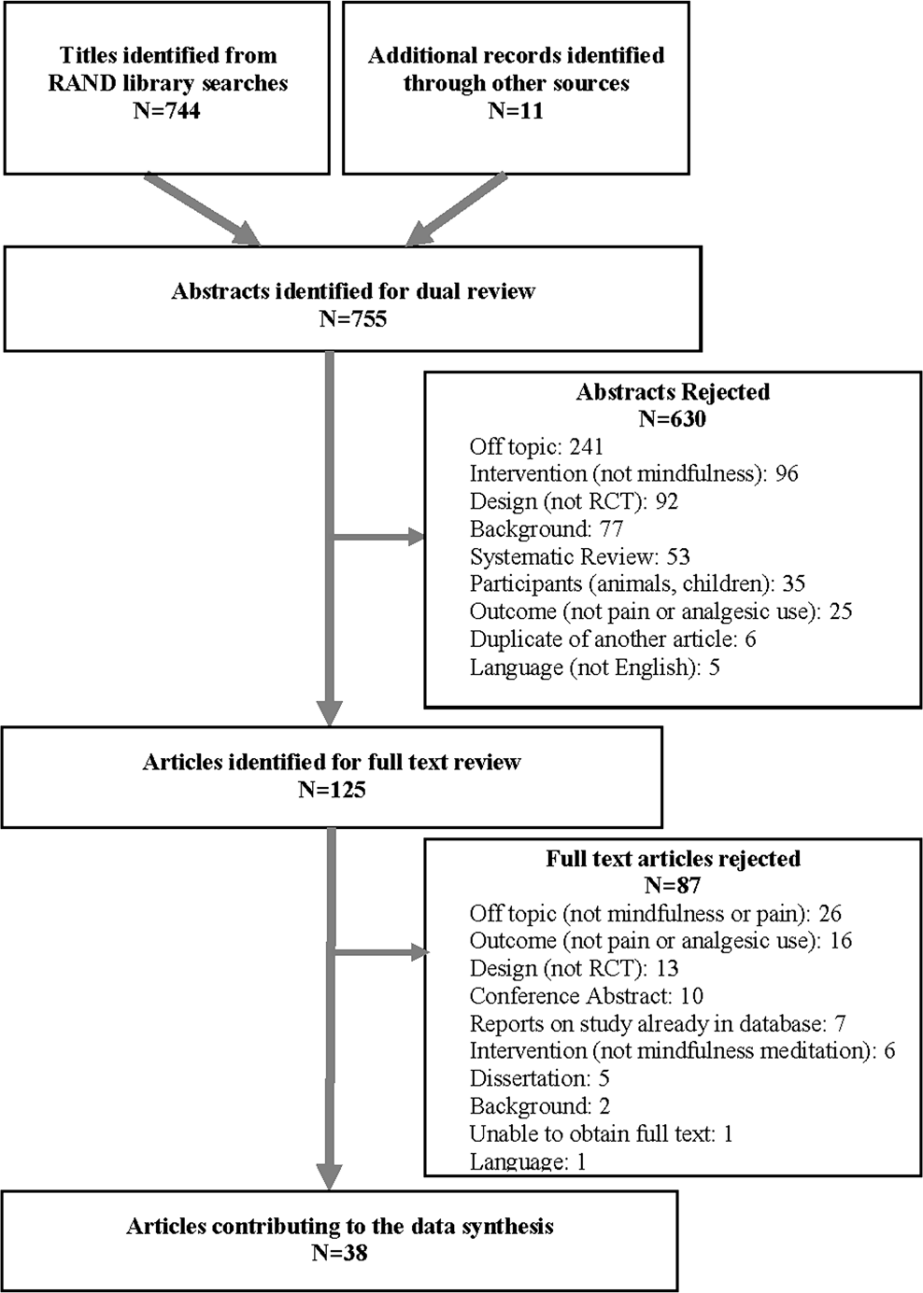
Literature flow diagram

We identified 744 citations through searches of electronic databases and 11 additional records identified through other sources (see Fig. 1). Full texts were obtained for 125 citations identified as potentially eligible by two independent reviewers; 38 RCTs met inclusion criteria. Details of study characteristics are displayed in Table 1 and effects for individual studies are displayed in Table 2.

**Table 1:** Characteristics of included studies

Study	Sample size	Location	Source of pain	% male	Age (M (SD))	Intervention	Comparators	Quality rating
Astin et al. [53]	128	USA	Fibromyalgia	0.7	48 (10.6)	MBSR and Qigong for 8 weeks	Education support group	Poor
Bakhshani et al. [61]	40	Middle East	Migraine, headache	35.1	Intervention, 30 (9.08); control, 31 (9.57)	MBSR for 8 weeks with TAU	TAU	Poor
Banth and Ardebil [60]	88	Middle East	Back pain	0	40.3(8.2)	MBSR for 8 weeks with TAU	TAU	Poor
Brown and Jones [54]	40	Europe	Fibromyalgia, rheumatoid arthritis, other musculoskeletal	26	Intervention, 48 (10); control, 45 (12)	Mindfulness-based pain management program: breath awareness, body awareness, gentle movement, pain management, compassion training for 8 weeks	TAU	Poor
Cash et al. [39]	91	USA	Fibromyalgia	0	Not reported	MBSR for 8 weeks	Waitlist control group	Fair
Cathcart et al. [40]	58	Australia	Headache	37	Intervention, 46 (13.10); control, 45 (14.2)	Brief mindfulness-based therapy, based on MBSR and MBCT for 3 weeks	Waitlist control group	Fair
Cherkin et al. [38]	342	USA	Back pain	34.3	49 (12.3)	MBSR for 8 weeks with TAU	CBT with TAU or TAU alone	Good
Davis and Zautra [42]	79	USA	Fibromyalgia	2	46; range = 22–81	Mindful social emotional regulation internet intervention in 12 modules for 6 weeks	Healthy lifestyle tips via internet	Fair
Day et al. [41]	36	USA	Migraine, headache	11	42 (12.0)	MBCT for 8 weeks adapted for headache pain with TAU	Waitlist control group	Fair
Dowd et al. [43]	124	Europe	Headache, back pain, osteoarthritis, fibromyalgia, nerve pain, neuropathy	10	45 (12.25)	MBCT computerized: included audio-recorded meditation, psychoeducation, mindfulness, and a cognitive and behavioral change for 6 weeks	Psychoeducation	Fair
Esmer et al. [55]	40	USA	Back pain, leg pain	56	Intervention, 55 (11.2); control, 58 (9.5)	MBSR for 8 weeks with TAU	TAU	Poor
Fjorback et al. [32]	120	Europe	Bodily distress syndrome	20	Intervention, 38 (9); control, 40 (8)	MBSR for 8 weeks with TAU	Enhanced TAU of 2-h specialist medical care and brief CBT	Good
Fogarty et al. [30]	51	New Zealand	Rheumatoid arthritis	12	Intervention, 52 (12); control, 55 (13)	MBSR for 8 weeks and TAU	TAU	Good
Garland et al. [44]	115	USA	Osteoarthritis, fibromyalgia	32	48 (14)	MORE: multimodal intervention of mindfulness, CBT, positive psychology for 8 weeks with TAU	Support group with TAU	Fair
Gaylord et al. [45]	75	USA	Irritable bowel syndrome	0	Intervention, 45 (12.55); control, 41 (14.68)	Mindfulness training tailored for IBS population for 8 weeks with TAU	TAU and support group	Fair
Jay et al. [50]	112	Europe	Musculoskeletal pain	0	Intervention, 45.5 (9.0); control, 47.6 (8.2)	Mindfulness pain and stress workplace program for 10 weeks	TAU	Fair
Johns et al. [37]	71	USA	Cancer	9.9	Intervention, 56 (9.9); control, 56 (12.7)	MBSR for 8 weeks	Psychoeducation support group	Good
Kanter et al. [62]	20	USA	Interstitial cystitis, bladder pain syndrome	0	Intervention, 46 (15.2); control, 44 (13.9)	MBSR for 8 weeks with TAU	TAU	Poor
Kearney et al. [51]	55	USA	Gulf War illness	85.5	Intervention,51 (6.8); control, 48 (7.4)	MBSR for 8 weeks with TAU	TAU	Fair
la Cour and Petersen [46]	109	Europe	Varied, non-specific pain	15	Intervention, 47 (12.42); control, 49 (12.20)	MBSR: Standard program modified for chronic pain patients for 8 weeks with co-intervention TAU	Waitlist, TAU	Fair
Lengacher et al. [52]	322	USA	Cancer	0	56.6 (9.7)	MBSR modified for breast cancer patients for 6 weeks with TAU	TAU	Fair
Ljotsson et al. [33]	85	Europe	Irritable bowel syndrome	15	35 (9.4)	MBCT protocol via Internet for IBS group treatment for 10 weeks	Online discussion forum	Good
Ljotsson et al. [34]	195	Europe	Irritable bowel syndrome	21	39 (11.1)	MBCT protocol via internet for IBS group treatment for 10 weeks	Online stress management program	Good
Meize-Grochowski et al. [56]	31	USA	Postherpetic neuralgia	44	Overall, 72 (9.6)	MBSR: 1 h instruction focusing breathing while seated comfortably, daily meditation using CD, phone call reminders, daily journal, for 6 weeks with TAU	TAU	Poor
Morone et al. [47]	37	USA	Back pain	43	Intervention, 74 (6.1); controls, 76 (5.0)	Modified MBSR: (1) the body scan; (2) sitting practice; (3) walking meditation for 8 weeks	Waitlist controls	Fair
Morone et al. [57]	40	USA	Back pain	37	Intervention, 78(7.1); control, 73(6.2)	Modified MBSR: (1) the body scan; (2) sitting practice; (3) walking meditation for 8 weeks; Over the counter and prescribed medications	Over the counter and prescribed medications	Poor
Morone et al. [36]	282	USA	Back pain	33.7	74.5 (6.6)	MBSR for 8 weeks	Health education program	Good
Omidi and Zargar [58]	66	Middle East	Headache	20	Intervention, 35 (2.41); control, 32 (3.2)	MBSR for 8 weeks	TAU	Poor
Parra-Delgado and Latorre-Postigo [31]	33	Europe	Fibromyalgia	0	53 (10.08)	MBCT for 12 weeks with TAU	TAU	Good
Plews-Ogan et al. [59]	30	USA	Musculoskeletal pain	23	47 (NR)	MBSR for 8 weeks	TAU (this group used in analysis) or massage	Poor
Rahmani and Talepasand [63]	24	Middle East	Cancer	0	Intervention, 43 (3.07); control, 45 (3.28)	MBSR and group conscious yoga for 8 weeks	No treatment	Poor
Schmidt et al. [48]	177	Europe	Fibromyalgia	0	53 (9.6)	Modified MBSR: mindfulness, yoga, and social interaction topics for 8 weeks	Waitlist (this group used in analysis), relaxation and stretching support group	Fair
Teixeira [64]	22	USA	Diabetic peripheral neuropathy	25	75 (10.8)	Modified MBSR: mindfulness meditation instruction and compact disk 5 days/week over a 4-week period for 4 weeks	Nutritional information and food diary	Poor
Wells et al. [49]	19	USA	Migraine	11	Intervention, 46 (17); control, 45 (12)	MBSR for 8 weeks	TAU	Fair
Wong [65]	100	Asia	Unspecified	NR	NR	MBSR for 8 weeks	Multidisciplinary education program	Poor
Wong [28]	100	Asia	Unspecified	NR	48 (7.84)	MBSR for 8 weeks	Multidisciplinary pain intervention	Good
Zautra et al. [29]	144	USA	Rheumatoid arthritis	32	Men, 62 (NR); women, 51 (NR)	Mindfulness meditation based on MBSR and emotion regulation therapy offered in sessions and home practice for 8 weeks	Education group (this group used in analysis), cognitive behavioral therapy	Good
Zgierska et al. [35]	35	USA	Back pain	20	51.8 (9.7)	MBCT-manualized program 6 days/week for 8 weeks with TAU	TAU and opioid therapy	Good

*Note*. *Age (M (SD))* age mean (standard deviation, range, or not reported), *CBT* cognitive behavioral therapy, *IBS* irritable bowel syndrome, *MBCT* mindfulness-based cognitive therapy, *MBSR* mindfulness-based stress reduction, *MORE* mindfulness-oriented recovery enhancement, *NR* not reported, *TAU* treatment as usual

**Table 2:** Effects for individual studies

Study	Outcome	Measure	% pain change Tx Grp	% pain change Ctrl Grp	SMD (95 % CI)	Follow-up (week)
Astin et al. [53]	Pain	SF-36 pain score	−31.58 %	−34.39 %	0.02 (−0.47, 0.5)	14
			−28.79 %	−35.03 %	−0.04 (−0.52, 0.45)	24
			−23.22 %	−29.94 %	−0.05 (−0.54, 0.43)	8
	Depression	BDI	–	–	0.15 (−0.35, 0.64)	24
Bakhshani et al. [61]	Pain		Significant improvement in pain, quality of life; sample size of groups at follow-up not reported			
	Quality of life					
Banth and Ardebil [60]	Pain	MPQ	−47.92 %	−11.62 %	2.5 (1.94, 3.07)	12
	Quality of life	SF-12 mental health	–	–	1.11 (0.65, 1.56)	8
			–	–	1.49 (1.01, 1.97)	12
		SF-12 physical health	–	–	1.34 (0.88, 1.81)	8
			–	–	1.86 (1.36, 2.37)	12
Brown and Jones [54]	Pain	Laser pain unpleasantness	9.26 %	6.78 %	0.24 (−0.51, 0.98)	24
	Quality of life, mental	SF-36 mental composite	–	–	1.16 (0.36, 1.96)	24
	Quality of life, physical	SF-36 physical composite	–	–	−0.42 (−1.17, 0.33)	24
Cash et al. [39]	Pain	VAS	−4.26 %	−5.92 %	0 (−0.42, 0.41)	16
			−11.31 %	−1.01 %	0.32 (−0.1, 0.74)	8
Cathcart et al. [40]	Pain	Headache intensity	−4.42 %	−11.16 %	0.08 (−0.52, 0.69)	8
Cherkin et al. [38]	Pain		Significant reductions in pain and disability; Reported only adjusted data			
	Disability					
Davis and Zautra [42]	Pain	Pain	−0.93 %	−1.52 %	−0.24 (−0.69, 0.2)	6
Day et al. [41]	Pain	BPI intensity	−11.98 %	−6.53 %	−0.01 (−0.66, 0.65)	8
Dowd et al. [43]	Pain	Average pain	1.26 %	−11.77 %	0 (−0.36, 0.35)	6
			7.18 %	1.71 %	−0.19 (−0.54, 0.17)	30
Esmer et al. [55]	Pain	VAS	−29.74 %	−0.82 %	0.3 (−0.5, 1.1)	12
	Disability	Roland-Morris disability	–	–	0.90 ( 0.06 , 1.74 )	12
Fjorback et al. [32]	Pain	SF-36 bodily pain	−31.25 %	−7.38 %	0.15 (−0.23, 0.53)	12
			−44.12 %	−12.08 %	0.23 (−0.18, 0.63)	36
			−34.93 %	−31.54 %	−0.1 (−0.51, 0.31)	60
	Quality of life, mental	SF-36 mental composite	–	–	−0.04 (−0.42, 0.34)	60
	Quality of life, physical	SF-36 physical composite	–	–	0.22 (−0.16, 0.61)	60
Fogarty et al. [30]	Pain			Significant reduction reported; no usable data as only reported change within group		
Garland et al. [44]	Pain	BPI	−12.32 %	11.11 %	0.76 (0.38, 1.14)	20
			−10.66 %	4.01 %	0.57 (0.19, 0.94)	8
Gaylord et al. [45]	Pain	Pain severity	−42.96 %	−14.73 %	0.53 (0.06, 0.99)	20
			−35.83 %	−5.36 %	0.54 (0.08, 1)	8
	Depression	BSI-18 depression	–	–	0.03 (−0.42, 0.49)	20
	Quality of life, general	IBS quality of life	–	–	0.25 (−0.21, 0.7)	20
Jay et al. [50]	Pain		Significant reduction reported; no usable data as only reported differences			
Johns et al. [37]	Pain	BPI	−35.19 %	−30.90 %	−0.07 (−0.54, 0.41)	24
Kanter et al. [62]	Pain	VAS	−16.95 %	−19.30 %	−0.14 (−1.05, 0.77)	8
	Quality of life	SF-12 MCS			0.7 (−0.24, 1.64)	8
		SF-12 PCS			−0.02 (−0.93, 0.89)	8
Kearney et al. [51]	Pain	MPQ	−23.87 %	−5.67 %	0.41 (−0.13, 0.94)	24
	Depression	PHQ	–	–	0.34 (−0.2, 0.87)	8
			–	–	0.47 (−0.07, 1)	24
la Cour and Petersen [46]	Pain	BPI	−1.05 %	−6.77 %	−0.16 (−0.53, 0.22)	8
	Depression	HADS	–	–	0.37 (−0.01, 0.75)	8
	Quality of life, mental	SF-36 mental composite	–	–	0.53 (0.15, 0.91)	8
	Quality of life, physical	SF-36 physical composite	–	–	0.00 (−0.38, 0.38)	8
Lengacher et al. [52]	Pain	BPI	−25.92 %	−10.63 %	0.02 (−0.2, 0.25)	12
	Depression	CES-D score	–	–	0.12 (−0.11, 0.35)	6
			–	–	0.04 (−0.18, 0.27)	12
	Quality of life	QoL MOS SF-36	–	–	−0.05 (−0.28, 0.17)	6
			–	–	0.01 (−0.21, 0.24)	12
Ljotsson et al. [33]	Pain	Total pain	−46.15 %	0.00 %	0.64 (0.19, 1.08)	10
	Depression	MADRS-S	–	–	0.43 (−0.02, 0.87)	10
	Quality of life, general	IBS quality of life	–	–	0.95 (0.49, 1.41)	10
	Disability	Sheehan disability scale	–	–	0.47 ( 0.02, 0.91)	10
Ljotsson et al. [34]	Pain			Significant reduction IBS pain/discomfort; no usable data as did not report pain measure	24	
	Depression	HADS	–	–	0 (−0.28, 0.28)	24
	Quality of life, general	IBS quality of life	–	–	0.51 (0.22, 0.8)	24
Meize-Grochowski et al. [56]	Pain	SF MPQ—total pain	−8.57 %	−4.17 %	−0.48 (−1.25, 0.28)	2
		SF MPQ—total pain	−25.71 %	−12.50 %	−0.31 (−1.07, 0.45)	8
	Depression	CES-D	–	–	0.32 (−1.08, 0.44)	8
	Quality of life, mental	Emotional well being	–	–	0.07 (−0.69, 0.82)	8
	Quality of life, physical	Average physical subscales	–	–	−0.02 (−0.77, 0.74)	8
Morone et al. [47]	Pain	SF MPQ	−11.61 %	3.29 %	0.23 (−0.42, 0.88)	8
	Quality of life, mental	SF-36 mental composite	–	–	0.22 (−0.43, 0.86)	8
	Quality of life, physical	SF-36 physical composite	–	–	0.11 (−0.53, 0.76)	8
	Disability	Roland-Morris disability	–	–	0.23 (−0.42, 0.87)	8
Morone et al. [57]	Pain	SF MPQ—total pain	−22.44 %	−26.71 %	−0.04 (−0.7, 0.63)	24
		SF MPQ—total pain	−26.28 %	−29.19 %	−0.01 (−0.68, 0.65)	8
Morone et al. [36]	Pain	Numeric pain rating—average	−13.64 %	0.95 %	0.22 (−0.01, 0.46)	24
	Quality of life	SF-36 global health composite	–	–	0.17 (−0.06, 0.41)	8
			–	–	0.03 (−0.2, 0.26)	24
		SF-36 physical health composite	–	–	0.17 (−0.06, 0.4)	8
			–	–	0 (−0.23, 0.23)	24
Plews-Ogan et al. [59]	Pain	Pain unpleasantness vs. TAU	−7.46 %	−8.70 %	0.02 (CI, −1.04, 1.07)	12
		Pain unpleasantness vs. massage	−7.46 %	−25.35 %	−0.16 (−1.19, 0.88)	12
		Pain unpleasantness vs. TAU	−16.42 %	−13.04 %	0.07 (CI, −0.99, 1.13)	4
		Pain unpleasantness vs. massage	−16.42 %	−12.68 %	−0.11 (CI, −0.92, 1.14)	4
		Pain unpleasantness vs. TAU	−13.43 %	−1.45 %	0.17 (CI, −0.89, 1.23)	8
		Pain unpleasantness vs. massage	−13.43 %	−39.44 %	−0.3 (CI, −1.34, 0.74)	8
	Quality of Life, mental	SF-12 mental composite	–	–	0.67 (−0.42, 1.75)	12
Rahmani and Talepasand [63]	Pain	Global quality symptoms—pain	−26.52 %	11.11 %	1.85 (0.89, 2.8)	16
			−44.89 %	−1.85 %	3.24 (2.02, 4.46)	8
	Quality of life, general	Global quality total score	–	–	1.18 (0.32, 2.05)	16
Schmidt et al. [48]	Pain	Pain perception scale vs. waitlist	−13.19 %	−6.90 %	0.17 (−0.2, 0.55)	16
		Pain perception scale vs. active	−13.19 %	−7.40 %	0.15 (−0.22, 0.53)	16
		Pain Perception Scale vs. waitlist	−11.87 %	−8.00 %	0.08 (−0.3, 0.45)	8
		Pain perception scale vs. active	−11.87 %	−4.86 %	0.22 (−0.16, 0.6)	8
	Depression	CES-D	–	–	0.1 (−0.27, 0.48)	16
	Quality of Life, General	QoL profile for chronically ill	–	–	0.26 (−0.12, 0.63)	16
Teixeira [64]	Pain	NeuroQoL pain	Missing baseline mean	0.14 (−0.74, 1.01)	4	
	Quality of life, general	NeuroQoL overall	–	–	0.79 (−0.12, 1.7)	4
Wells et al. [49]	Pain	Headache severity	−25.00 %	0.00 %	0.99 (0.04, 1.95)	12
		Headache severity	−27.27 %	8.33 %	1.5 (0.48, 2.51)	8
	Depression	PHQ	–	–	0.59 (−0.33, 1.51)	8
	Quality of life, general	Migraine-specific QoL	–	–	−0.43 (−1.34, 0.48)	8
Wong [65]	Pain			Significant pain decrease; no usable data		
Wong [28]	Pain			No significant effect; no usable data		
Zautra et al. [29]	Pain	Pain vs. education	−14.49 %	−17.70 %	0.22 (−0.2, 0.63)	8
		Pain vs. cognitive behavior therapy	−14.49 %	−14.34 %	0.56 (0.16, 0.96)	8
	Depression	Depressive symptoms	–	–	0.28 (−0.13, 0.7)	8
Zgierska et al. [35]	Pain		Significant pain decrease; no follow-up data available			

*Note*. *BDI* Beck depression inventory, *BPI* brief pain index; *BS b*rief symptom inventory, *CES-D* The Center for Epidemiological Studies-Depression Scale, *CI* confidence interval, *MADRS* Montgomery-Asberg Depression Rating Scale, *HADS* Hospital Anxiety and Depression Scale; *MPQ* McGill Pain Questionnaire, *NPS* Neuropathic Pain Scale, *NeuroQoL* Quality of Life in Neurological Disorders; *PHQ* Patient Health Questionnaire, *QoL* quality of life, *SF-36* Short-Form Health Survey 36, *SF MPQ* Short-Form McGill Pain Questionnaire, *SMD* standardized mean difference, *TAU* treatment as usual, *VAS* Visual Analog Scale

In total, studies assigned 3536 participants; sample sizes ranged from 19 to 342. Fifteen studies reported an a priori power calculation with targeted sample size achieved, ten studies did not report information about a power calculation, and three studies were unclear in the reporting of a power calculation. Ten studies noted there was insufficient power; the authors considered these pilot studies. The majority of the studies were conducted in North America or Europe. The mean age of participants ranged from 30 (SD, 9.08) to 78 years (SD, 7.1. Eight studies included only female participants.

Medical conditions reported included fibromyalgia in eight studies and back pain in eight studies. (Categories are not mutually exclusive; some studies included patients with different conditions.) Osteoarthritis was reported in two studies and rheumatoid arthritis in three. Migraine headache was reported in three studies and another type of headache in five studies. Three studies reported irritable bowel syndrome (IBS). Eight studies reported other causes of pain and three studies did not specify a medical condition or source of chronic pain.

The total length of the interventions ranged from 3 to 12 weeks; the majority of interventions (29 studies) were 8 weeks in length. Twenty-one studies were conducted on mindfulness-based stress reduction (MBSR) and six on mindfulness-based cognitive therapy (MBCT). Eleven additional studies reported results on other types of mindfulness training. Thirteen RCTs provided the mindfulness intervention as monotherapy, and eighteen utilized a mindfulness intervention as adjunctive therapy, specifying that all participants received this in addition to other treatment such as medication. Seven of the studies were unclear as to whether the mindfulness intervention was monotherapy or adjunctive therapy. Nineteen RCTs used treatment as usual as comparators, thirteen used passive comparators, and ten used education/support groups as comparators. Beyond these common comparators, one study each used stress management, massage, a multidisciplinary pain intervention, relaxation/stretching, and nutritional information/food diaries as comparators; two studies used cognitive-behavioral therapy. Several studies had two comparison arms.

### Study Quality and Risk of Bias

The study quality for each included study is displayed in Table 1. Eleven studies obtained a “good” quality rating [28–38]. Fourteen studies were judged to be of fair quality, primarily due to being unclear in some aspects of the methods [39–52]. Thirteen studies were judged to be poor; ten primarily due to issues with completeness of reporting outcome data such as inadequate or missing intention to treat (ITT) analysis and/or less than 80 % follow-up [53–62] and three due to unclear methods [63–65]. Details of the quality ratings and risk of bias for each included study is displayed in Electronic Supplementary Material 1.

### Measures

Studies reported patient pain measures such as the Visual Analog Scale, the SF-36 pain subscale, and McGill Pain Questionnaire. Secondary outcome measures included depression symptoms (e.g., Beck Depression Inventory, Patient Health Questionnaire), physical and mental health-related quality of life (e.g., SF-36 mental and physical components), and functional impairment/disability (e.g., Roland-Morris Disability Questionnaire, Sheehan Disability Scale).

### Chronic Pain Treatment Response

Thirty RCTs reported continuous outcome data on scales assessing chronic pain [29, 31–33, 36, 39–49, 51–60, 62–64, 66].

Eight studies met screening inclusion criteria but did not contribute to the meta-analysis because they did not report poolable data [28, 30, 34, 35, 38, 50, 61, 65]. Their study characteristics are displayed in Table 1, and study level effects along with the reasons they were not in pooled analyses are displayed in Table 2.

**Fig. 2:**
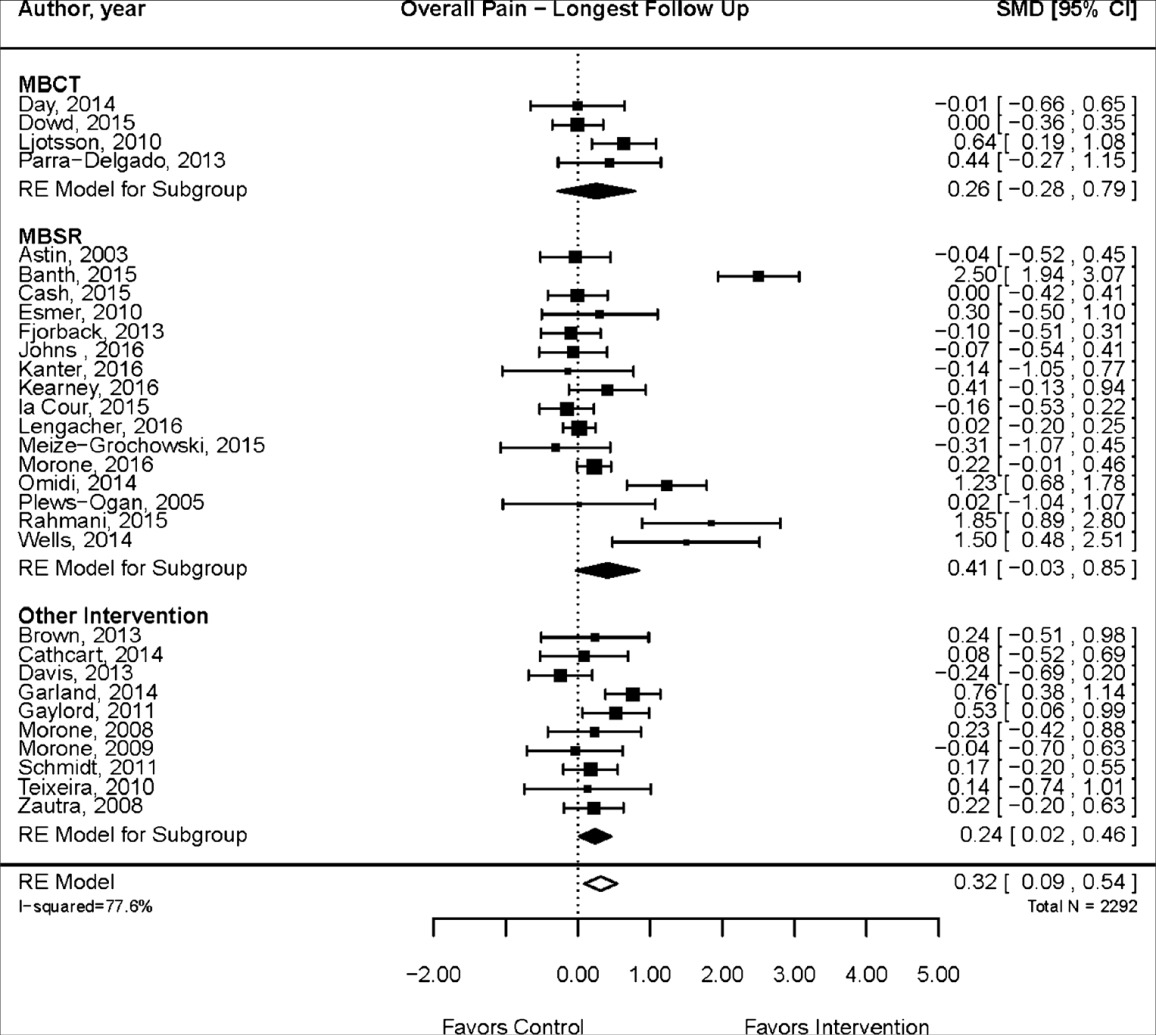
Mindfulness meditation effects on chronic pain

Pain scales and comparators varied from study to study. The median follow-up time was 12 weeks, with a range of 4 to 60 weeks. Figure 2 displays the results of meta-analysis using data at the longest follow-up for each study. The pooled analysis indicates a statistically significant effect of mindfulness meditation compared with treatment as usual, passive controls, and education/support groups (SMD, 0.32; 95 % CI, 0.09, 0.54; 30 RCTs). Substantial heterogeneity was detected (*I* ^2^ = 77.6 %). There was no evidence of publication bias (Begg’s *p* = 0.26; Egger’s test *p* = 0.09). To investigate whether the treatment estimate is robust when excluding poor-quality studies and to explore the possible source of the substantial heterogeneity, we conducted a sensitivity analysis including only fair or good quality studies. The improvement remained significant, the effect size was smaller (SMD, 0.19; 95 % CI, 0.03, 0.34; 19 RCTs), and there was less heterogeneity (*I* ^2^ = 50.5 %). Meta-regressions showed that changes in pain outcomes in good- (*p* = 0.42) and fair-quality (*p* = 0.13) studies were not significantly different from changes in poor-quality studies.

In subgroup analyses, the effect was not statistically significant at 12 weeks or less (SMD, 0.25; 95 % CI, −0.13, 0.63; 15 RCTs; *I* ^2^ = 82.6 %) but was significant for follow-up periods beyond 12 weeks (SMD, 0.31; 95 % CI, 0.04, 0.59; 14 RCTs, *I* ^2^ = 69.0 %). Begg’s test was not statistically significant (*p* = 0.16) but Egger’s test showed evidence of publication bias (*p* = 0.04). The quality of evidence that mindfulness meditation is associated with a decrease in chronic pain compared with control is low overall and for both short- and long-term follow-up due to inconsistency, heterogeneity, and possible publication bias. A detailed table displays the quality of evidence for findings for each major outcome in Electronic Supplementary Material 2.

In order to present clinically meaningful results, we calculated the percent change in pain symptoms from baseline to follow-up for mindfulness meditation and comparison groups for each study and displayed findings in Table 2. We then calculated the overall weighted mean percent change for mindfulness meditation groups versus comparison groups for effects of meditation for pain at longest follow-up. The mean percent change in pain for meditation groups was −0.19 % (SD, 0.91; min, −0.48; max, 0.10) while the mean percent change in pain for control groups was −0.08 % (SD, 0.74; min, −0.35; max, 0.11). The *p* value for the difference between groups was significant (*p* = 0.0031).

### Depression

Depression outcomes were reported in 12 RCTs [29, 31, 33, 34, 45, 46, 48, 49, 51–53, 56]. Overall, meditation significantly lowered depression scores as compared with treatment as usual, support, education, stress management, and waitlist control groups (SMD, 0.15; 95 % CI, 0.03, 0.26; 12 RCTs; *I* ^2^ = 0 %). No heterogeneity was detected. The quality of evidence was rated as high due to lack of heterogeneity, consistent study results, and precision of effect (small confidence intervals).

### Quality of Life

Sixteen studies reported mental health-related quality of life; the effect of mindfulness meditation was significant in the pooled analysis as compared with treatment as usual, support groups, education, stress management, and waitlist controls (SMD, 0.49; 95 % CI, 0.22, 0.76; *I* ^2^, 74.9 %). [32–34, 45–49, 52, 54, 56, 59, 60, 62–64]. Sixteen studies measured physical health-related quality of life [32–34, 36, 45–49, 52, 54, 56, 60, 62–64]. Pooled analyses showed a significant effect of mindfulness meditation as compared with treatment as usual, support groups, education, stress management, and waitlist controls (SMD, 0.34; 95 % CI, 0.03, 0.65; *I* ^2^, 79.2 %). Both quality-of-life analyses detected substantial heterogeneity, and the quality of evidence was rated as moderate for mental health (small confidence intervals, more consistent results) and low for physical health-related quality of life.

### Functional Impairment (Disability Measures)

Four studies reported poolable disability scores from the Roland-Morris Disability Questionnaire and the Sheehan Disability Scale [33, 36, 47, 55]. The difference between the mindfulness and comparison groups in follow-up was not statistically significant (SMD, 0.30; 95 % CI, −0.02, 0.62; *I* ^2^ = 1.7 %), although the results approached significance. No heterogeneity was detected. The quality of evidence was rated low due to imprecision and small total sample size.

### Analgesic Use

Only four studies reported use of analgesics as an outcome. In a study of MBSR for treatment of chronic pain due to failed back surgery syndrome [55], at 12-week follow-up, the analgesic medication logs of the intervention group documented a decrease in analgesic use compared with those in the control group (−1.5 (SD = 1.8) vs. 0.4 (SD = 1.1), *p* = <0.001). A study of mindfulness meditation and cognitive-behavioral therapy vs. usual care for low back pain [35] reported that the mean morphine equivalent dose (mg/day) of opioids was not significantly different between groups at both 8 and 26 weeks. Likewise, a trial of MBSR for back pain [38] found no significant difference between groups in self-reported use of pain medication. Finally, a trial of mindfulness-oriented recovery enhancement (MORE) for chronic pain of various etiologies [44] found intervention participants significantly more likely to no longer meet criteria for opioid use disorder immediately following treatment (*p* = 0.05); however, these effects were not sustained at 3-month follow-up.

### Adverse Events

Only 7 of the 38 included RCTs reported on adverse events. Four stated no adverse events occurred [36, 47, 50, 57]; one described that two participants experienced temporary strong feelings of anger toward their pain condition and two of the participants experienced greater anxiety [46]; two studies recorded mild side effects from yoga and progressive muscle relaxation [35, 38].

### Study Characteristic Moderators

Meta-regressions were run to determine if changes in pain outcomes systematically differed by several subcategories. There was no difference in effect on pain between MBSR (16 studies) and MBCT (4 studies; *p* = 0.68) or other types of mindfulness interventions (10 studies; *p* = 0.68). When comparing MBSR (16 studies) to all other interventions (14 studies), there was also no difference in effect (*p* = 0.45). As stated in more detail above, medical conditions reported included fibromyalgia, back pain, arthritis, headache, and irritable bowel syndrome (IBS). Meta-regressions did not suggest differences between headache (six studies) and other conditions (*p* = 0.93), back pain (eight studies) and other conditions (*p* = 0.15), and fibromyalgia (eight studies) and other conditions (*p* = 0.29). Gender composition (% male) had no association with effect on pain (*p* = 0.26). The total length of the intervention program ranged from 3 to 12 weeks (mean was 8 weeks). Meta-regression did not suggest differences between high-frequency interventions and medium- (*p* = 0.16) or low-frequency (*p* = 0.44) interventions. No systematic difference in effect on pain between adjunctive therapy and monotherapy (*p* = 0.62) or between adjunctive therapy and interventions where this was unclear (*p* = 0.10) was found. Finally, there was no systematic difference in effect whether the comparator was treatment as usual, waitlist, or another intervention (*p* = 0.21).

## Discussion

In sum, mindfulness meditation was associated with a small effect of improved pain symptoms compared with treatment as usual, passive controls, and education/support groups in a meta-analysis of 30 randomized controlled trials. However, there was evidence of substantial heterogeneity among studies and possible publication bias resulting in a low quality of evidence. The efficacy of mindfulness meditation on pain did not differ systematically by type of intervention, medical condition, or by length or frequency of intervention. Mindfulness meditation was associated with statistically significant improvement in depression, physical health-related quality of life, and mental health-related quality of life. Quality of evidence was high for depression, moderate for mental health-related quality of life, and low for physical health-related quality of life. Only four studies reported on change in analgesic use; results were mixed. Adverse events in the included RCTs were rare and not serious, but the vast majority of studies did not collect adverse events data.

This review has several methodological strengths: an a priori research design, duplicate study selection and data abstraction of study information, a comprehensive search of electronic databases, risk of bias assessments, and comprehensive quality of evidence assessments used to formulate review conclusions. One limitation is that we did not contact individual study authors; results reported in the review are based on published data. We excluded conference abstracts which do not contain enough data to evaluate study quality. In addition, we included only studies published in English.

The included studies had many limitations. Thirteen of the thirty-eight studies were rated as poor quality, primarily due to lack of ITT, poor follow-up, or poor reporting of methods for randomization and concealment of allocation. The authors of ten studies reported inadequate statistical power to detect differences in pain outcomes between mindfulness meditation and the comparator; the authors considered these pilot studies. Ten other studies did not report a power calculation. Sample sizes were small; 15 studies randomized fewer than 50 participants.

More well-designed, rigorous, and large RCTs are needed in order to develop an evidence base that can more decisively provide estimates of its effectiveness. Studies should enroll samples large enough to detect statistical differences in outcomes and should follow-up with participants for 6 to 12 months in order to assess the long-term effects of meditation. Adherence to mindfulness practice and simultaneous use of other therapies should be monitored frequently. Intervention characteristics, including the optimal dose, have also not yet conclusively been established. In order to detect intervention specific effects, studies need to have attention-matched controls. Smaller trials may be conducted to answer these questions. Other outcomes that were outside the scope of this review may be important to explore. As the impact of mindfulness may be related to the appraisal of the pain, it may be useful for future trials to focus primary outcomes on symptoms associated with pain such as quality of life, pain-related interference, pain tolerance, analgesic, and related issues such as opioid craving. Future publications on RCTs of mindfulness meditation should adhere to Consolidated Standards of Reporting Trials (CONSORT) standards.

Only three RCTs attributed minor adverse events to mindfulness meditation. However, only 7 of the 38 included RCTs mentioned whether adverse events were monitored and collected. Thus quality of evidence for adverse events reported in RCTs is inadequate for a comprehensive assessment. Given published reports of adverse events during meditation, including psychosis [67], future trials should actively collect adverse events data. In addition, a systematic review of observational studies and case reports would shed additional light on adverse events during mindfulness meditation.

Further research examining the effect of mindfulness meditation on chronic pain should also focus on better understanding whether there is a minimum frequency or duration of meditation practice for it to be effective. While recent studies have yielded similar positive effects of mindfulness for pain, these effects tend to be small to medium and based on a body of evidence that is, at best, of moderate quality. A potential way to advance research on chronic pain would be to improve intervention and control group descriptions, identify different effects of various components of complex interventions, and work toward a standard criterion for assessing therapeutic gain [68]. Head-to-head trials that compare mindfulness interventions of a similar category but with variations in components or dose may be helpful to tease out the most effective elements of these interventions [69].

Similar to previous reviews in this area, we conclude that while mindfulness meditation interventions showed significant improvements for chronic pain, depression, and quality of life, the weaknesses in the body of evidence prevent strong conclusions. The available evidence did not yield consistent effects for pain outcomes, and few studies were available for forms of mindfulness meditation other than MBSR. Quality of evidence for the efficacy of mindfulness interventions in reducing chronic pain is low. There was higher quality evidence of the efficacy of mindfulness meditation on depression and mental health-related quality-of-life outcomes. This review is consistent with previous reviews concluding that more well-designed, rigorous, and large RCTs are needed in order to develop an evidence base that can more decisively provide estimates of the efficacy of mindfulness meditation for chronic pain. In the meantime, chronic pain continues to pose a tremendous burden on society and individuals. A novel therapeutic approach for chronic pain management such as mindfulness meditation would likely be welcomed by patients suffering from pain.

## Electronic Supplementary Material

ESM 1 (DOCX 33 kb)

ESM 2 (DOCX 26 kb)

## References

[1] Chou R, Turner JA, Devine EB (2015). The effectiveness and risks of long-term opioid therapy for chronic pain: a systematic review for a National Institutes of Health pathways to prevention workshop. Annals of Internal Medicine.

[2] Institute of Medicine: Relieving pain in America: A blueprint for transforming prevention, care, education, and research (report brief). www.iom.edu/relievingpain*. 2011.* 22553896

[3] Department of Veterans Affairs Department of Defense: VA/DoD clinical practice guideline for management of opioid therapy for chronic pain. May 2010.

[4] Chiesa A, Serretti A (2011). Mindfulness-based interventions for chronic pain: a systematic review of the evidence. Journal of Alternative and Complementary Medicine.

[5] Kabat-Zinn J, Lipworth L, Burney R (1985). The clinical use of mindfulness meditation for the self-regulation of chronic pain. Journal of Behavioral Medicine.

[6] MARC: *UCLA Mindfulness Awareness Research Center.* Accessed May 29, 2015. http://marc.ucla.edu/default.cfm

[7] Brewer JA, Garrison KA (2014). The posterior cingulate cortex as a plausible mechanistic target of meditation: findings from neuroimaging. Ann NY Acad Sci.

[8] Boccia M, Piccardi L, Guariglia P: The meditative mind: a comprehensive meta-analysis of MRI studies. Biomed Res Int 2015, Article ID 419808*:*1–11. 10.1155/2015/419808 PMC4471247 26146618

[9] Chiesa A, Serretti A (2014). Are mindfulness-based interventions effective for substance use disorders? A systematic review of the evidence. Substance Use and Misuse.

[10] de Souza IC, de Barros VV, Gomide HP (2015). Mindfulness-based interventions for the treatment of smoking: a systematic literature review. Journal of Alternative and Complementary Medicine.

[11] Goyal M, Singh S, Sibinga EM (2014). Meditation programs for psychological stress and well-being: a systematic review and meta-analysis. JAMA Intern Med.

[12] Kozasa EH, Tanaka LH, Monson C (2012). The effects of meditation-based interventions on the treatment of fibromyalgia. Curr Pain Headache Rep.

[13] Cramer H, Haller H, Lauche R, Dobos G (2012). Mindfulness-based stress reduction for low back pain. A systematic review. BMC Complementary and Alternative Medicine.

[14] Reiner K, Tibi L, Lipsitz JD (2013). Do mindfulness-based interventions reduce pain intensity? A critical review of the literature. Pain Medicine.

[15] Lauche R, Cramer H, Dobos G, Langhorst J, Schmidt S (2013). A systematic review and meta-analysis of mindfulness-based stress reduction for the fibromyalgia syndrome. Journal of Psychosomatic Research.

[16] Lakhan SE, Schofield KL (2013). Mindfulness-based therapies in the treatment of somatization disorders: a systematic review and meta-analysis. PloS One.

[17] Merkes M (2010). Mindfulness-based stress reduction for people with chronic diseases. Aust J Prim Health.

[18] Lee C, Crawford C, Hickey A (2014). Mind-body therapies for the self-management of chronic pain symptoms. Pain Medicine.

[19] Bawa FL, Mercer SW, Atherton RJ (2015). Does mindfulness improve outcomes in patients with chronic pain? Systematic review and meta-analysis. British Journal of General Practice.

[20] Higgins J, Green S: Cochrane handbook for systematic reviews of interventions, version 5.1.0; 2011.

[21] US Preventive Services Task Force: *US Preventive Services Task Force Procedure Manual*. Rockville, MD: Agency for Healthcare Research and Quality; 2008.

[22] The Lewin Group and ECRI Institute: Management of dyslipidemia: Evidence synthesis report. Clinical practice guideline. 2014.

[23] Hartung J (1999). An alternative method for meta-analysis. Biometrical Journal.

[24] Hartung J, Knapp G (2001). A refined method for the meta-analysis of controlled clinical trials with binary outcome. Statistics in Medicine.

[25] Sidik K, Jonkman JN (2006). Robust variance estimation for random effects meta-analysis. Computational Statistics & Data Analysis.

[26] Balshem H, Helfand M, Schunemann HJ (2011). GRADE guidelines: 3. Rating the quality of evidence. Journal of Clinical Epidemiology.

[27] Egger M, Davey Smith G, Schneider M, Minder C (1997). Bias in meta-analysis detected by a simple, graphical test. BMJ.

[28] Wong SY, Chan FW, Wong RL (2011). Comparing the effectiveness of mindfulness-based stress reduction and multidisciplinary intervention programs for chronic pain: a randomized comparative trial. Clinical Journal of Pain.

[29] Zautra AJ, Davis MC, Reich JW (2008). Comparison of cognitive behavioral and mindfulness meditation interventions on adaptation to rheumatoid arthritis for patients with and without history of recurrent depression. Journal of Consulting and Clinical Psychology.

[30] Fogarty FA, Booth RJ, Gamble GD, Dalbeth N, Consedine NS (2015). The effect of mindfulness-based stress reduction on disease activity in people with rheumatoid arthritis: a randomised controlled trial. Annals of the Rheumatic Diseases.

[31] Parra-Delgado M, Latorre-Postigo JM (2013). Effectiveness of mindfulness-based cognitive therapy in the treatment of fibromyalgia: a randomised trial. Cognitive Therapy and Research.

[32] Fjorback LO, Arendt M, Ornbol E (2013). Mindfulness therapy for somatization disorder and functional somatic syndromes: randomized trial with one-year follow-up. Journal of Psychosomatic Research.

[33] Ljotsson B, Falk L, Vesterlund AW (2010). Internet-delivered exposure and mindfulness based therapy for irritable bowel syndrome--a randomized controlled trial. Behaviour Research and Therapy.

[34] Ljotsson B, Hedman E, Andersson E (2011). Internet-delivered exposure-based treatment vs. stress management for irritable bowel syndrome: a randomized trial. American Journal of Gastroenterology.

[35] Zgierska AE, Burzinski CA, Cox J, et al. 2016 Mindfulness meditation and cognitive behavioral therapy intervention reduces pain severity and sensitivity in opioid-treated chronic low back pain: pilot findings from a randomized controlled trial. Pain Medicine 10.1093/pm/pnw006 PMC5063022 26968850

[36] Morone NE, Greco CM, Moore CG (2016). A mind-body program for older adults with chronic low back pain: a randomized clinical trial. JAMA Intern Med.

[37] Johns SA, Brown LF, Beck-Coon K, et al. 2016 Randomized controlled pilot trial of mindfulness-based stress reduction compared to psychoeducational support for persistently fatigued breast and colorectal cancer survivors. Supportive Care in Cancer 10.1007/s00520-016-3220-4 PMC5221754 27189614

[38] Cherkin DC, Sherman KJ, Balderson BH (2016). Effect of mindfulness-based stress reduction vs cognitive behavioral therapy or usual care on back pain and functional limitations in adults with chronic low back pain: a randomized clinical trial. JAMA.

[39] Cash E, Salmon P, Weissbecker I (2015). Mindfulness meditation alleviates fibromyalgia symptoms in women: results of a randomized clinical trial. Annals of Behavioral Medicine.

[40] Cathcart S, Galatis N, Immink M, Proeve M, Petkov J (2014). Brief mindfulness-based therapy for chronic tension-type headache: a randomized controlled pilot study. Behavioural and Cognitive Psychotherapy.

[41] Day MA, Thorn BE, Ward LC (2014). Mindfulness-based cognitive therapy for the treatment of headache pain: a pilot study. Clinical Journal of Pain.

[42] Davis MC, Zautra AJ (2013). An online mindfulness intervention targeting socioemotional regulation in fibromyalgia: results of a randomized controlled trial. Annals of Behavioral Medicine.

[43] Dowd H, Hogan MJ, McGuire BE (2015). Comparison of an online mindfulness-based cognitive therapy intervention with online pain management psychoeducation: a randomized controlled study. Clinical Journal of Pain.

[44] Garland EL, Manusov EG, Froeliger B (2014). Mindfulness-oriented recovery enhancement for chronic pain and prescription opioid misuse: results from an early-stage randomized controlled trial. Journal of Consulting and Clinical Psychology.

[45] Gaylord SA, Palsson OS, Garland EL (2011). Mindfulness training reduces the severity of irritable bowel syndrome in women: results of a randomized controlled trial. American Journal of Gastroenterology.

[46] la Cour P, Petersen M (2015). Effects of mindfulness meditation on chronic pain: a randomized controlled trial. Pain Medicine.

[47] Morone NE, Greco CM, Weiner DK (2008). Mindfulness meditation for the treatment of chronic low back pain in older adults: a randomized controlled pilot study. Pain.

[48] Schmidt S, Grossman P, Schwarzer B (2011). Treating fibromyalgia with mindfulness-based stress reduction: results from a 3-armed randomized controlled trial. Pain.

[49] Wells RE, Burch R, Paulsen RH (2014). Meditation for migraines: a pilot randomized controlled trial. Headache.

[50] Jay K, Brandt M, Hansen K (2015). Effect of individually tailored biopsychosocial workplace interventions on chronic musculoskeletal pain and stress among laboratory technicians: randomized controlled trial. Pain Physician.

[51] Kearney DJ, Simpson TL, Malte CA (2016). Mindfulness-based stress reduction in addition to usual care is associated with improvements in pain, fatigue, and cognitive failures among veterans with gulf war illness. American Journal of Medicine.

[52] Lengacher CA, Reich RR, Paterson CL, et al. (2016) Examination of broad symptom improvement resulting from mindfulness-based stress reduction in breast cancer survivors: A randomized controlled trial. Journal of Clinical Oncology 10.1200/JCO.2015.65.7874 PMC5012660 27247219

[53] Astin JA, Berman BM, Bausell B (2003). The efficacy of mindfulness meditation plus qigong movement therapy in the treatment of fibromyalgia: a randomized controlled trial. Journal of Rheumatology.

[54] Brown CA, Jones AK (2013). Psychobiological correlates of improved mental health in patients with musculoskeletal pain after a mindfulness-based pain management program. Clinical Journal of Pain.

[55] Esmer G, Blum J, Rulf J, Pier J (2010). Mindfulness-based stress reduction for failed back surgery syndrome: a randomized controlled trial. Journal of the American Osteopathic Association.

[56] Meize-Grochowski R, Shuster G, Boursaw B (2015). Mindfulness meditation in older adults with postherpetic neuralgia: a randomized controlled pilot study. Geriatric Nursing (New York, N.Y.).

[57] Morone NE, Rollman BL, Moore CG, Li Q, Weiner DK (2009). A mind-body program for older adults with chronic low back pain: results of a pilot study. Pain Medicine.

[58] Omidi A, Zargar F (2014). Effect of mindfulness-based stress reduction on pain severity and mindful awareness in patients with tension headache: a randomized controlled clinical trial. *Nursing and Midwifery*. Studies.

[59] Plews-Ogan M, Owens JE, Goodman M, Wolfe P, Schorling J (2005). A pilot study evaluating mindfulness-based stress reduction and massage for the management of chronic pain. Journal of General Internal Medicine.

[60] Banth S, Ardebil MD (2015). Effectiveness of mindfulness meditation on pain and quality of life of patients with chronic low back pain. Int J Yoga.

[61] Bakhshani NM, Amirani A, Amirifard H, Shahrakipoor M (2016). The effectiveness of mindfulness-based stress reduction on perceived pain intensity and quality of life in patients with chronic headache. Glob J Health Sci.

[62] Kanter G, Komesu YM, Qaedan F, et al.: Mindfulness-based stress reduction as a novel treatment for interstitial cystitis/bladder pain syndrome: A randomized controlled trial. Int Urogynecol J. 2016. 10.1007/s00192-016-3022-8 PMC5067184 27116196

[63] Rahmani S, Talepasand S (2015). The effect of group mindfulness—based stress reduction program and conscious yoga on the fatigue severity and global and specific life quality in women with breast cancer. Medical Journal of the Islamic Republic of Iran.

[64] Teixeira E (2010). The effect of mindfulness meditation on painful diabetic peripheral neuropathy in adults older than 50 years. Holistic Nursing Practice.

[65] Wong SY (2009). Effect of mindfulness-based stress reduction programme on pain and quality of life in chronic pain patients: a randomised controlled clinical trial. Hong Kong Medical Journal. Xianggang Yi Xue Za Zhi.

[66] Fjorback LO, Arendt M, Ornbol E, Fink P, Walach H (2011). Mindfulness-based stress reduction and mindfulness-based cognitive therapy: a systematic review of randomized controlled trials. Acta Psychiatrica Scandinavica.

[67] Kuijpers HJ, van der Heijden FM, Tuinier S, Verhoeven WM (2007). Meditation-induced psychosis. Psychopathology.

[68] Morley S, Williams A (2015). New developments in the psychological management of chronic pain. Canadian Journal of Psychiatry. Revue Canadienne de Psychiatri.

[69] Kerns RD, Burns JW, Shulman M (2014). Can we improve cognitive-behavioral therapy for chronic back pain treatment engagement and adherence? A controlled trial of tailored versus standard therapy. Health Psychology.

