# Supplementary material for: Mindfulness Meditation for Chronic Pain: Systematic Review and Meta-analysis
Source: Ann Behav Med. 2016 Sep 22;51(2):199–213. doi: 10.1007/s12160-016-9844-2 (PMC5368208; doi:10.1007/s12160-016-9844-2)
Supplement: Supplementary file 1 — (DOCX 33 kb) [file 12160_2016_9844_MOESM1_ESM.docx]

Electronic Supplementary Material 1. Quality and Risk of Bias of Included Studies

| Study ID | Random Sequence Generation (selection bias) | Allocation Concealment (selection bias) | Blinding of Participants and Personnel (performance bias) | Blinding of Outcome Assessors (detection bias) | Completeness of Reporting Outcome Data (attrition bias) | Selective Outcome Reporting (reporting bias | Other Biases | | | | | USPSTF Quality Ratinga |
| --- | --- | --- | --- | --- | --- | --- | --- | --- | --- | --- | --- | --- |
|  |  |  |  |  |  |  | Unequal Distribution Among Groups of Potential Confounders at Baseline | Crossovers or Contamination Between Groups | Equal, Reliable, and Valid Outcome Measurement | Clear Definitions of Interventions | ITT Analysis |  |
| Astin, Berman, et al., 2003 | Low risk | Low risk | High risk | Unclear risk | High risk | Unclear risk | No | No | Yes | Yes | No | Poor |
| Bakhshani et al., 2016 | Unclear risk | Unclear risk | High risk | High risk | Unclear risk | Unclear risk | No | No | No | Yes | Yes | Poor |
| Banth and Ardebil, 2015 | Unclear risk | Unclear risk | Unclear risk | Low risk | Unclear risk | Unclear risk | Unclear | No | No | Yes | Unclear | Poor |
| Brown and Jones, 2013 | Unclear risk | Unclear risk | High risk | Unclear risk | High risk | Unclear risk | Unclear | No | No | Yes | No | Poor |
| Cash et al., 2015 | Low risk | Unclear risk | High risk | Unclear risk | Low risk | Low risk | No | No | Yes | Yes | Yes | Fair |
| Cathcart et al., 2014 | Unclear risk | Low risk | High risk | Low risk | Low risk | Low risk | No | No | Yes | Yes | No | Poor |
| Cherkin et al., 2016 | Low risk | Low risk | High risk | Low risk | Low risk | Low risk | Unclear | No | No | Yes | No | Good |
| Davis and Zautra, 2013 | Low risk | Low risk | High risk | High risk | Low risk | Low risk | No | No | Yes | Yes | Yes | Fair |
| Day et al., 2014 | Low risk | High risk | High risk | High risk | Low risk | Unclear risk | No | No | Yes | Yes | Yes | Fair |
| Dowd et al., 2015 | Low risk | Low risk | High risk | High risk | Low risk | Unclear risk | No | No | Yes | Yes | Yes | Fair |
| Esmer et al., 2010 | Unclear risk | Unclear risk | High risk | High risk | High risk | Unclear risk | No | No | Yes | Yes | Yes | Poor |
| Fjorback et al., 2013 | Low risk | Low risk | High risk | Unclear risk | Low risk | Unclear risk | No | No | Yes | Yes | Yes | Good |
| Fogarty et al., 2015 | Unclear risk | Unclear risk | High risk | Low risk | Low risk | Unclear risk | No | No | Yes | Yes | Yes | Good |
| Garland et al., 2014 | Low risk | Low risk | High risk | Low risk | Low risk | Unclear risk | No | No | Yes | Yes | Yes | Fair |
| Gaylord et al., 2011 | Low risk | Low risk | High risk | Low risk | Low risk | Low risk | No | No | Yes | Yes | Yes | Fair |
| Jay et al., 2015 | Low risk | Unclear risk | High risk | Low risk | Low risk | Low risk | No | No | No | Yes | No | Fair |
| Johns et al., 2016 | Low risk | Low risk | High risk | Unclear risk | Low risk | Low risk | No | No | No | Yes | No | Good |
| Kanter et al., 2016 | Low risk | Low risk | Unclear risk | High risk | Unclear risk | Low risk | No | No | No | Yes | Unclear | Poor |
| Kearney et al., 2016 | Unclear risk | Unclear risk | High risk | Low risk | Low risk | Low risk | No | No | No | Yes | No | Fair |
| la Cour and Petersen, 2015 | Low risk | Low risk | High risk | High risk | Low risk | Unclear risk | Yes | No | Yes | Yes | Yes | Fair |
| Lengacher et al., 2016 | Low risk | Unclear risk | High risk | High risk | Low risk | Low risk | Yes | No | No | Yes | No | Fair |
| Ljotsson, Falk, et al., 2010 | Low risk | Low risk | High risk | High risk | Low risk | Unclear risk | Unclear | No | Yes | Yes | Yes | Good |
| Ljotsson, Hedman, et al., 2011 | Low risk | Low risk | High risk | High risk | Low risk | High risk | No | No | Yes | Yes | Yes | Good |
| Meize-Grochowski et al., 2015 | Unclear risk | Unclear risk | High risk | High risk | High risk | Unclear risk | Unclear | No | Yes | Yes | No | Poor |
| Morone, Greco, and Weiner, 2008 | Low risk | Low risk | High risk | Unclear risk | Low risk | Unclear risk | No | No | Yes | Yes | Yes | Fair |
| Morone et al., 2009 | Low risk | Low risk | High risk | Low risk | High risk | Unclear risk | Yes | No | Yes | Yes | No | Poor |
| Morone et al., 2016 | Low risk | Low risk | High risk | Low risk | Low risk | Low risk | No | No | No | Yes | No | Good |
| Omidi and Zargar, 2014 | Unclear risk | Unclear risk | Unclear risk | Unclear risk | High risk | Low risk | Yes | No | Yes | Yes | No | Poor |
| Parra-Delgado and Latorre-Postigo, 2013 | Low risk | Unclear risk | High risk | Unclear risk | Low risk | Low risk | No | No | Yes | Yes | Yes | Good |
| Plews-Ogan et al., 2005 | Low risk | Unclear risk | High risk | High risk | High risk | Unclear risk | No | No | Yes | Yes | No | Poor |
| Rahmani and Talepasand, 2015 | Unclear risk | Unclear risk | High risk | Unclear risk | Low risk | Low risk | Yes | No | Yes | Yes | Yes | Poor |
| Schmidt et al., 2011 | Unclear risk | Unclear risk | Low risk | Low risk | Low risk | High risk | No | No | Yes | Yes | Yes | Fair |
| Teixeira, 2010 | Unclear risk | Unclear risk | High risk | Unclear risk | Low risk | Low risk | Unclear | No | Yes | Yes | Yes | Poor |
| Wells et al., 2014 | Low risk | Low risk | High risk | Unclear risk | Low risk | Low risk | Unclear | No | Yes | No | Yes | Fair |
| Wong, 2009 | Unclear risk | Unclear risk | High risk | High risk | Unclear risk | Unclear risk | No | No | Yes | Yes | Unclear | Poor |
| Wong et al., 2011 | Low risk | Low risk | High risk | High risk | Low risk | Unclear risk | No | No | Yes | Yes | Yes | Good |
| Zautra et al., 2008 | Low risk | Unclear risk | High risk | High risk | Low risk | Unclear risk | No | No | Yes | Yes | Yes | Good |
| Zgierska et al., 2016 | Low risk | Low risk | High risk | High risk | Low risk | Low risk | Yes | No | No | Yes | No | Good |
| a USPSTF = U.S. Preventive Services Task Force. The USPSTF criteria (U.S. Preventive Services Task Force, 2008) for study quality involve assessment of various factors related to the internal validity of the study. “Good” is the highest ranking, which involves comparable groups with low attrition, with outcomes being reliably and validly measured and analyzed. “Fair” is the next highest rating and involves studies with one or a few potential concerns (e.g., some though not major differences between groups exist at follow-up), though intention-to-treat analysis was performed. “Poor” is the lowest ranking and involves studies with one or more “fatal flaws” (e.g., no ITT analysis). | | | | | | | | | | | | |
