# Supplementary material for: Mindfulness Meditation for Chronic Pain: Systematic Review and Meta-analysis
Source: Ann Behav Med. 2016 Sep 22;51(2):199–213. doi: 10.1007/s12160-016-9844-2 (PMC5368208; doi:10.1007/s12160-016-9844-2)
Supplement: Supplementary file 2 — (DOCX 26 kb) [file 12160_2016_9844_MOESM2_ESM.docx]

Electronic Supplementary Material 2. Summary of Findings and Quality of Evidence

| Outcome | Study Design (number of RCTs and participants | Findings (direction and magnitude of effect) | Study Limitations  (study quality;  risk of bias) | Inconsistency | Indirectness | Imprecision | GRADE of Evidence for Outcome |
| --- | --- | --- | --- | --- | --- | --- | --- |
|  |  |  |  |  |  |  |  |
| Pain: Longest follow-up | 30 RCTs,  2,292 participants | SMD 0.32 (CI 0.09, 0.54), favors mindfulness meditation | Mixed quality;  effect smaller (SMD 0.19) when poor quality RCTs excluded | Inconsistent; substantial heterogeneity | Direct | Precise | Low |
| Pain: 0–12 weeks follow-up | 15 RCTs,  1,017 participants | SMD 0.25 (CI -0.13, 0.63) n.s. | Mixed quality | Inconsistent; substantial heterogeneity | Direct | Imprecise | Low |
| Pain: >12 weeks follow-up | 14 RCTs,  1,256 participants | SMD 0.31 (CI 0.04, 0.59), favors mindfulness meditation | Mixed quality;  possible publication bias | Inconsistent; substantial heterogeneity | Direct | Precise | Low |
| Depression | 12 RCTs,  1,178 participants | SMD 0.15 (CI 0.03, 0.26), favors mindfulness meditation | Majority good or  fair quality | Consistent; no heterogeneity | Direct | Precise | High |
| Physical health-related quality of life | 16 RCTs,  1,551 participants | SMD 0.34 (CI 0.03, 0.65), favors mindfulness meditation | Mixed quality | Inconsistent; substantial heterogeneity | Direct | Precise | Low |
| Mental health-related quality of life | 16 RCTs,  1,283 participants | SMD 0.49 (CI 0.22, 0.76), favors mindfulness meditation | Mixed quality | Consistent; substantial heterogeneity | Direct | Precise | Moderate |
| Functional impairment/disability measures | 4 RCTs,  425 participants | SMD 0.30 (CI −0.02, 0.62), n.s. | Mixed quality | Consistent, no heterogeneity | Direct | Imprecise | Low |
| NOTE: CI=Confidence intervals; n.s.=not significant; RCT=Randomized controlled trial; SMD=standardized mean difference. | | | | | | | |
